# Conservative treatment with mouthwashes followed by tongue photo biomodulation therapy in Covid-19: a case report

**DOI:** 10.1186/s13256-022-03519-z

**Published:** 2022-10-06

**Authors:** Saira Chaughtai, Zeeshan Chaughtai, Arif Asif

**Affiliations:** Department of Medicine, Hackensack Meridian Jersey Shore University Medical Center—Hackensack-Meridian School of Medicine, Neptune, NJ 07753 USA

**Keywords:** COVID-19, Oral manifestations, Mouth rinses, Systemic rash, Photo biomodulation therapy

## Abstract

**Background:**

Oral manifestations of coronavirus disease 2019 (COVID-19), including ulcers, herpetiform lesions, macules, and petechiae, among others, are becoming increasingly recognized, but there is little guidance on their treatment. Reported cases have described treatment with various mouthwashes containing antivirals, antifungals, antibiotics, anesthetics, or steroids. Our case report is unique in that we provide guidance on the judicious use of these medications, followed by photobiomodulation therapy if the manifestations are treatment resistant.

**Case presentation:**

We describe a 30-year-old Caucasian woman who tested positive for COVID-19 after developing nasal congestion and cough. Ten days after testing positive, she developed a systemic rash on her extremities and torso. At the same time, she developed swelling of the tongue lasting 1 hour, with subsequent appearance of oral lesions that resembled geographic tongue. She also had an irritable sensation on her tongue and some mild loss of sense of taste. We opted for conservative therapy, including mouth rinses containing lidocaine to be used every 6 hours. The patient used the mouth rinse therapy for 1 month and experienced a 90% improvement in her oral lesions and tongue sensitivity. However, she had repeated flares every 3 weeks over a 6-month period, and the steroid mouthwash achieved incomplete resolution. After three sessions of photobiomodulation therapy, she had no further flares or tongue sensitivity and the lesions healed.

**Conclusions:**

The implication of our report is that we promote the judicious use of topical antibiotics, antivirals, antifungals, and steroids for when they are indicated. We propose lidocaine-containing mouth rinses and steroid mouthwash as an initial, symptomatic treatment regimen for ‘COVID-19 tongue.’ If there is failure of resolution, we recommend photobiomodulation therapy.

## Introduction

Coronavirus disease 2019 (COVID-19) blew the world away with its rapid and alarming spread across the globe. The COVID-19 pandemic was accompanied by new manifestations of this viral illness, including oral manifestations. Patients infected with severe acute respiratory syndrome coronavirus 2 (SARS-CoV-2) presented with complaints of various oral symptoms, including ulcers and erosions of the tongue. In this article, we review the current literature on the treatment of ‘COVID-19 tongue’ and describe a case of COVID-19 tongue that we treated. When we reviewed the literature, we could not find clear guidance on treatment of COVID-19 tongue. We initiated conservative treatment consisting of mouth rinses of lidocaine and steroids. When that failed to achieve complete resolution, we utilized photobiomodulation therapy with success.

## Case presentation

**Fig. 1:**
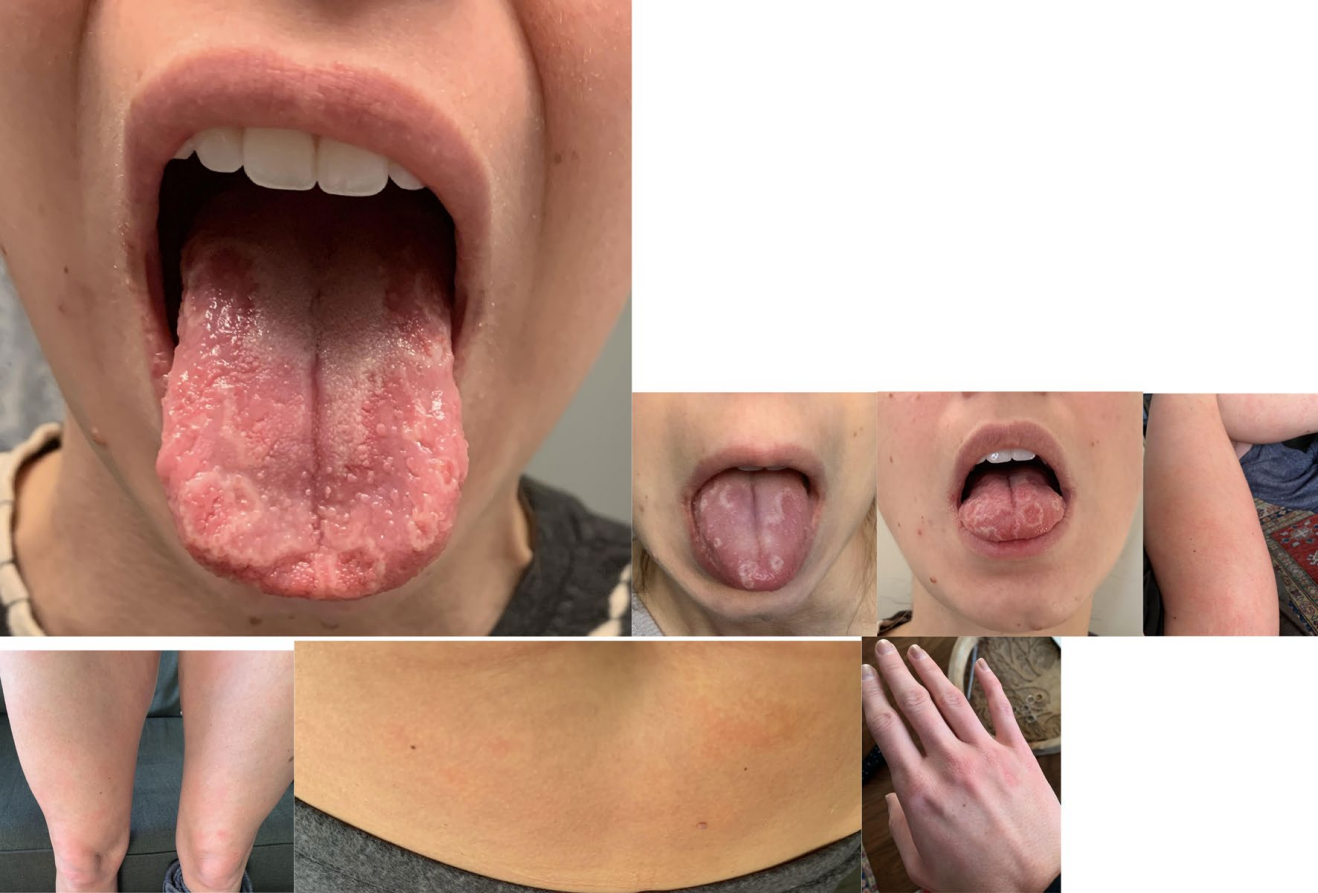
Pictures of patient’s tongue, extremities, torso, and hand

A previously healthy 30-year-old Caucasian woman presented to her primary care office with complaints of COVID-19. She developed symptoms after her husband returned from international travel. She initially had symptoms of an upper respiratory tract infection, including nasal congestion and cough. She had fatigue and shortness of breath on ambulation. Ten days after her initial symptoms, she developed an urticarial rash on her abdomen, legs, and hands (Fig. 1). The rash was transient and reappeared twice before disappearing. The rash only lasted hours on each occasion. The patient has no past medical history and takes no medications. She does not drink alcohol, smoke cigarettes, or use drugs. She is married and lives at home with her husband, works as a wedding planner, has no children, and has never been pregnant.

She also complained of tongue swelling on the tenth day after developing COVID-19. The swelling went away after 1 hour on its own, but she then developed geographic tongue with the appearance of tongue papillae that were inflamed and some papillae that were flattened (Fig. 1). Her tongue had a sensation of sensitivity in the areas interspersed between areas of regular mucosa, and she had some mild loss of sense of taste.

In the office, her blood pressure was 117/71 mmHg, heart rate was 90 beats per minute, temperature was 98.4 °F, respirations were 14 per minute, and pulse oximetry was 100% on room air. On further examination, she had a geographic appearance to the tongue, with denuding of regions of tongue papillae with an irregular appearance. Her abdomen and extremities showed no signs of rash at the time of the clinical examation. Her neurological examination showed intact motor and sensory strength, normal heart sounds, and lungs that were clear to auscultation; the rest of her examation was normal. Her white blood cell count was 4100/μL (normal range 3800–11,000/μL), hemoglobin was 13.8 (normal range: 13–17) gm/dL, and platelets were 248,000/μL (normal range: 140,000-450,000/μL). Creatinine level was 0.79 (normal range: 0.5–1.1) mg/dL, aspartate transaminase level was 18 (normal range: 10–30) U/L, and alanine transaminase level was 17 (normal range: 16-29) U/L. Her vitamin B12 level was 468 [normal range: 200–1100) pg/mL, and her serum folate was 10.3 (Normal range: > 5.4) ng/mL.

**Fig. 2:**
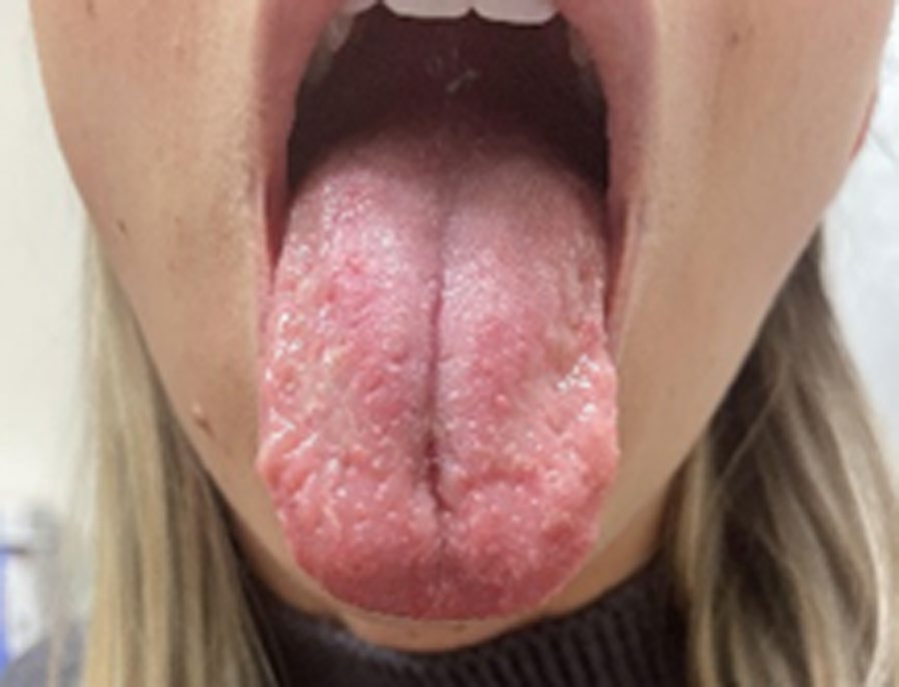
The patient’s tongue after receiving photobiomodulation therapy, showing healing

She was diagnosed as having COVID tongue and was started on a mouthwash containing diphenhydramine-lidocaine-aluminum-magnesium-simethicone-nystatin to be swished and swallowed orally at a dose of 5 mL every 6 hours. She subsequently completed 30 days of the mouthwash and achieved 90% improvement in the appearance of the geographic tongue. Over the next 6 months, she had multiple flares of tongue sensitivity and visually active tongue lesions about every 3 weeks, and she was prescribed dexamethasone 0.5mg/5mL to be swished and spit four times daily for 10 days. However, she did not achieve complete resolution of her tongue lesions. Six months after developing COVID tongue, she underwent three treatments of photobiomodulation therapy (PBMT) to her tongue with an interval of 24 hours between each session. At a follow-up 2 months after the PBMT, she had no further flares and her tongue lesions were healing (Fig. 2). Her systemic rash never returned, and she subsequently did well.

## Discussion

We present a case of COVID tongue in an otherwise healthy 30-year-old woman. She was initially treated with a lidocaine-containing mouthwash for 30 days and achieved 90% remission of her tongue symptoms. She further received a 10-day course of steroid mouthwash but was unable to achieve complete remission of her COVID tongue. After receiving PBMT to the tongue, she had no further COVID flares at the 2-month follow-up. Our case is unique in that we took a step-based approach to the treatment of COVID tongue, initially using a conservative therapy of lidocaine and steroids mouthwashes. When the conservative treatment was ineffective, we opted for PBMT which achieved full remission from further flares and healing of tongue lesions.

COVID-19-related oral manifestations are now becoming a recognized phenomenon of SARS-CoV-2 infection. Various descriptions of these manifestations have been reported, including erosions, ulcers, and depapillation of the tongue [1]. The authors of one study noted that older patients and those with more severe disease had worse oral manifestations [1]. In our case, however, our patient was a healthy young woman with no comorbidities and mild disease. It is very possible that the lack of oral manifestations in those with mild disease may represent underreporting due to quarantine. There have even been reports of lesions resembling erythema multiforme [2].

Oral lesions have been reported to be more likely to occur at the onset of disease in mild cases, and 1–3 weeks later in those with more severe disease [3]. This is again in contrast to our patient who had only mild disease and did not require hospitalization. She developed oral lesions 10 days after her initial symptoms of COVID-19. It has also been reported that oral manifestations represent coinfections due to immune deterioration or secondary events due to hospitalization, illness, or intubation [3, 4]. However, there was no evidence of coinfection in our patient and she did not have any secondary factors that led to her oral manifestations as she had only mild COVID-19 disease and remained at home for the duration of her disease.

The other varieties of oral manifestations observed are petechiae, herpetiform lesions, blisters, and macules [5]. The pathogenesis of oral lesions in COVID-19 is postulated to have multiple etiologies, including being the result of direct inflammation of the SARS-CoV-2 virus in the oral cavity, or of adverse effects of medications used to treat COVID-19 [5]. The immune dysregulation that COVID-19 causes will also lead to a breakdown in immunity, allowing opportunistic infections like candida to take hold [5]. ACE2 (angiotensin-converting enzyme 2) receptors are utilized by the SARS-CoV-2 virus to gain entry into the host [5]. The oral cavity contains many ACE2 receptors and this is considered the entryway to cause COVID-19-related inflammation of the oral cavity [5–7]. The infection of the salivary glands may be the reason why dysgeusia is a prominent symptom in COVID-19 [8, 9]. Both the tongue and salivary glands highly express ACE2 receptors [8–11]. The authors of a review found that altered taste occurred as the first symptom of COVID-19 in 11% of patients [12]. This manifestation of COVID-19 is believed to be a combination of olfactory neuron dysfunction and taste bud dysfunction, leading to the smell and taste disorders in COVID-19 [13].

Our patient presented with a geographic tongue, also known as erythema migrans [14]. There have been very few reports of geographic tongue associated with COVID-19 [14]. Geographic tongue consists of irregular areas of depapillation, and its incident in adults is 1–2% [14]. Geographic tongue is believed to be associated with elevated interleukin-6 levels, which is the same cytokine by which SARS-CoV-2 inflicts its damage [14]. Geographic tongue is not usually associated with viral infections and is idiopathic [15].

In most cases, the oral lesions heal within a couple of weeks. In a case series of 26 patients with oral ulcers associated with COVID-19, oral paracetamol and chlorhexidine washes were utilized, which helped with remission of patients in their 1 to 2 weeks of follow-up [16]. Another report showed oral lesions resolving within 3 weeks [17]. Some studies conducted in China have attempted to correlate severity degree and gross appearance of the tongue with severity of COVID-19 disease [18].

Our patient also had a systemic rash affecting her torso and extremities. MIS-c (multisystem inflammatory syndrome in children) is the form of COVID-19 manifesting in children. Interestingly, in children with MIS-c and rash, the outcomes are better, with fewer respiratory symptoms and fewer admissions to the intensive care unit [19]. Halepas *et al.* noted in their study of pediatric patients with oral lesions that there was a significant association of these lesions with systemic rash [20]. Our patient did have a relatively mild course of disease, with no requirement for any medication and only mild upper respiratory symptoms. In a study of children with MIS-c, 59% had a rash [21]. The incidence of rash in adult patients with COVID-19 is significantly lower, especially that of urticarial rash as in our patient. In one study, the incidence of rash was only 5% among 103 patients analyzed [22].

Our patient’s systemic urticarial rash appeared twice for a few hours before disappearing altogether. The appearance of her rash coincided with her tongue manifestations. Her tongue did swell for 1 hour prior to breaking out into a geographic appearance. This is possibly in line with the systemic inflammatory response mediated by cytokines. In one study, the incidence of tongue swelling in association with oral lesions of COVID-19 was reported to be 6% [23]. One report described a case of tongue swelling along with swelling of the floor of the mouth and bilateral neck swelling in a COVID-19 patient [24].

Various treatment options have been utilized, including mouth rinses, with steroids being reserved for severe cases [25]. Common mouth rinses used for this purpose contain various ingredients, such as antifungals, antibiotics, antivirals, chlorhexidine, hydrogen peroxide, hyaluronic acid, and anesthetics (for example, lidocaine) [25]. Topical steroids have been used as well [25]. In general, oral steroids and oral antifungals have been avoided except in cases where clinically indicated [25]. We propose that steroids be avoided except in severe, unremitting cases that have not achieved resolution of symptoms with conservative mouth rinses because steroids can cause opportunistic infections, such as candida, and can have side effects. We also recommend not utilizing antifungals, antivirals, or antibiotics unless the infections have a typical clinical appearance or if there is laboratory evidence of a viral, fungal, or bacterial infection. We recommend a good history and physical examination, in correlation with laboratory evidence to make a judicious decision regarding treatment. In our patient’s case, the lidocaine-containing mouthwash was used with some effect, resulting in 90% remission of her tongue geographic appearance. A 10-day course of steroid mouthwash was unable to cause complete remission of her symptoms of tongue sensitivity.

Garcez *et al.* described a case of COVID tongue treated with PBMT and the successful resolution of symptoms of edema and pain within 7 days of treatment [26]. Teixeira *et al.* further described a series of cases where PBMT led to relief of pain and orofacial symptoms of COVID-19 [27]. There was another reported case of the opportunistic infections candidiasis and herpes labialis present in a patient with COVID-19 [28]. The patient was treated with PBMT with resolution of the symptoms and without the need for systemic treatment for the opportunistic infections [28]. There is growing evidence of cases in which PBMT has helped in the resolution of COVID-19-related oral symptoms [29]. Our patient is another example of the success of PBMT as she had no further flares of her tongue lesions at the 2-month follow up after completing PBMT.

## Conclusion

COVID-19 has been associated with oral manifestations. These manifestations are postulated to be a direct result of inflammation and secondary due to breakdown in immunity, coinfections, and hypersensitivity to drugs utilized for COVID-19 treatment. We first treated our patient with COVID tongue with lidocaine and steroid mouthwashes and had good results, but not complete resolution of symptoms. We further treated her with PBMT, achieving healing and resolution of her flares. We propose a thoughtful and step-wise approach to treatment of oral lesions associated with COVID-19, including an initial conservative approach and use of PBMT in those patients failing to achieve resolution with a conservative approach.

## Data Availability

Data sharing not applicable to this article as no datasets were generated or analyzed during the current study.
